# Cervical Cancer Screening Cascade: A Framework for Monitoring Uptake and Retention Along the Screening and Treatment Pathway

**DOI:** 10.3390/curroncol32070407

**Published:** 2025-07-17

**Authors:** Sara Izadi-Najafabadi, Laurie W. Smith, Anna Gottschlich, Amy Booth, Stuart Peacock, Gina S. Ogilvie

**Affiliations:** 1 BC Cancer, Vancouver, BC V5Z 0B4, Canada; speacock@bccrc.ca; 2 Women’s Health Research Institute, BC Women’s Hospital, Vancouver, BC V6H 3N1, Canada; amy.booth@cw.bc.ca (A.B.); gina.ogilvie@bccdc.ca (G.S.O.); 3 Karmanos Cancer Institute, Wayne State University, Detroit, MI 48201, USA; hp7073@wayne.edu; 4 School of Population and Public Health, University of British Columbia, Vancouver, BC V6T 1Z3, Canada; 5 Faculty of Health Sciences, Simon Fraser University, Burnaby, BC V5A 1S6, Canada

**Keywords:** cervical cancer, screening cascade, HPV screening

## Abstract

**Background:** Cervical cancer is a major global health concern, causing approximately 350,000 deaths annually. It is also preventable through effective prevention and early detection. To facilitate elimination, the World Health Organization (WHO) set targets for HPV vaccination, screening, and treatment. Achieving these goals requires frameworks to monitor screening program performance. As many regions transition to HPV primary screening, a standardized Cervical Cancer Screening Cascade can track performance, identify gaps in follow-up, and optimize resource allocation. **Methods:** This paper introduces a structured cascade developed to monitor uptake, retention, and outcomes in HPV-based screening programs. The Cascade was created through collaboration between public health experts, clinicians, and researchers at the University of British Columbia (UBC), the Women’s Health Research Institute, and BC Cancer. **Results:** The Cascade outlines four phases: screening, triage, detection, and treatment. Each phase includes two substages: “uptake” and “results,” with an additional substage in screening (“invitation”). “Screening” assesses invitation effectiveness and participation. “Triage” tracks follow-up after a positive screen. “Detection” evaluates attendance at diagnostic appointments, and “Treatment” measures the treatment rate for those with precancerous lesions. **Conclusions:** The Cascade can guide emerging and existing HPV screening programs within Canada and other similarly resourced settings and serve as a benchmark tool for programs to assess their progress towards cervical cancer elimination.

## Simple Summary

Cervical cancer is largely preventable, yet it continues to cause hundreds of thousands of deaths worldwide. Effective screening and treatment are key to reducing this burden, especially with the shift to human papillomavirus (HPV) testing as the primary, more effective screening method. However, many individuals do not complete all recommended steps, such as follow-up testing or treatment after receiving a positive screening result. This paper introduces a structured framework—the Cervical Cancer Screening Cascade—to help cervical screening programs monitor the proportion of eligible individuals completing each stage of the screening and treatment process. By identifying where drop-off occurs in the cascade of care, this framework intends to improve care delivery, inform policy, and support global efforts to eliminate cervical cancer.

## 1. Introduction

Cervical cancer, the fourth most common cancer among women around the world, is a preventable disease, yet remains a global health concern [1]. In 2020, the World Health Organization (WHO) launched a global strategy to eliminate cervical cancer (i.e., incidence rate below 4 per 100,000) by the end of the century [2]. The strategy includes three key targets to be achieved by 2030: Pillar 1: 90% of girls fully vaccinated against human papillomavirus (HPV) by age 15; Pillar 2: 70% of women screened with a high-performance test by age 35 and again at 45, and; Pillar 3: 90% of women with cervical disease receiving appropriate treatment [2,3].

In alignment with the WHO strategy, the Canadian Partnership Against Cancer (CPAC) launched an action plan to eliminate cervical cancer in Canada by 2040 [4]. CPAC’s ‘90-90-90’ plan aims to have 90% of 17-year-olds vaccinated by 2025, 90% of eligible individuals up-to-date with HPV screening, and 90% of those requiring treatment with an appropriate follow-up plan by 2030 [4].

Elimination of cervical cancer is possible [2]; however, questions remain regarding the optimal strategies for achieving the recommended targets. The goal of cervical screening is to detect and treat cervical precancerous lesions, specifically cervical intraepithelial neoplasia (CIN). CIN grade 2 (CIN2), which represents moderate dysplasia, is commonly used as the threshold for treatment in many settings before lesions progress to invasive cancer [5]. However, for screening to be effective, and facilitate early detection and treatment of lesions, adherence to recommended follow-up is critical. Loss to follow-up after invitation, after screening, after reflex/triage testing, and before treatment significantly undermines overall health outcomes [6,7,8]. This is of particular concern in HPV primary screening programs with a self-screening option, where individuals who historically did not attend provider-collected cytology due to multiple barriers may not adhere to recommended follow-up screening and treatment [6,9,10], potentially significantly reducing the overall impact and benefits of HPV screening. Therefore, it is essential to closely monitor uptake and retention within screening and treatment programs, as well as related outcomes (e.g., precancer detection rate) to ensure the long-term effectiveness of screening interventions.

Jurisdictions around the world are in various stages of implementing HPV primary screening—a well-established, high-performance screening approach that is superior to cytology testing for detection of cervical precancer [11,12,13,14]. In addition, HPV testing can be performed on samples collected vaginally or cervically, providing new opportunities for enhancing the reach of cervical screening [15,16,17,18]. A Canadian modelling study demonstrated that increasing screening participation from 70% to 90% or increasing compliance with attendance at colposcopy could accelerate the elimination of cervical cancer by 2 years [19]. Other studies in high-income settings have also shown that cervical cancer can be eliminated within 10–15 years post-HPV screening implementation [20,21]. Given the global transition to HPV screening, development of a comprehensive and standard cascade of care is timely and important. A structured pathway, from identification of the screening-eligible population to diagnosis and treatment, ensures that each stage is clearly defined and monitored, which facilitates the identification of gaps in follow-up care, and allows for the development of interventions to address these gaps [22]. Efficient resource allocation is another benefit of a well-defined cascade, as it identifies stages with higher rates of loss to follow-up, allowing for resource redirection to those stages, to improve overall program effectiveness.

Cervical cancer screening cascades within specific programs or settings around the world have been previously described (See Table 1) [22,23,24,25,26]. These cascades delineate the various stages in cervical cancer screening and care, with a specific emphasis on confirming cervical cancer cases and implementing appropriate interventions based on results at each stage [22,23,24,25,26]. While common elements across these cascades include screening, provision of results, attendance at follow-up testing, and treatment if needed, each published cascade of care concentrates on a specific aspect of a program, population, or setting; for example, rural women in China [24], women living with HIV [22], or those receiving radiation therapy as treatment [25]. Therefore, these cascades are minimal in their generalizability to other screening programs. Furthermore, the stages and measures defined are not standardized, which hinders cross-program comparisons. Notably, none of these cascades were designed specifically for an HPV primary screening program.

Our goal is to develop and describe a Cervical Cancer Screening Cascade (“the Cascade”) to provide a shared framework for programs in high-resource settings to monitor uptake and retention across the screening pathway. Although this Cascade can be applied to a cytology screening approach, it has been developed with a focus on HPV primary screening, the Cascade will offer a framework for evaluating key outcomes such as program uptake and retention at each stage of detection and treatment of precancerous lesions. In addition, for programs transitioning from cytology to HPV screening, the Cascade enables comparison of previous cytology screening metrics to HPV primary screening outcomes, to comprehensively review uptake and retention through the transition.

A Cervical Cancer Screening Cascade can be utilized alongside the indicators proposed by WHO (90-70-90) and CPAC (90-90-90)—in particular, pillars two (screening) and three (attendance at follow-up)—to help quantify uptake, loss to follow-up, and attrition rates across the care spectrum, thereby affording the ability to address significant attrition points, identify the underlying causes, and guide interventions to retain women and individuals with a cervix (WIC) at each stage. In this paper, we will describe the Cervical Cancer Screening Cascade and provide practical steps for programs to evaluate HPV primary screening through implementation of the Cervical Cancer Screening Cascade.

## 2. The Cervical Cancer Screening Cascade Development

The Cervical Cancer Screening Cascade was developed through a collaborative process involving public health experts, clinicians, and researchers at the University of British Columbia, the Women’s Health Research Institute at BC Women’s Hospital, and BC Cancer in Vancouver, Canada. The process incorporated a review of existing publicly available cervical cancer screening cascades (Table 1) and extensive consultations to identify common challenges and gaps in program performance. The development was iterative, involving multiple rounds of refinement and modifications to the stages and corresponding metrics, with the goal of balancing simplicity, ease of implementation, and sufficient detail to ensure the Cascade’s effectiveness in guiding cervical cancer prevention initiatives. This collaborative approach ensured that the Cascade would be both practical and adaptable across different screening programs from a variety of regions.

To ensure consistency across resources, thus facilitating clearer communication and better alignment on global cervical cancer elimination efforts, whenever possible, definitions for each stage of the Cascade are adapted from the Improving Data for Decision Making in Global Cervical Cancer Programmes (IDCCP) report [27]. The WHO published this report in 2019 to enhance the quality and use of data in the fight to eliminate cervical cancer, particularly in low- and middle-income countries. It was developed to support global health programs in collecting accurate and timely data for better decision-making in cervical cancer prevention, screening, treatment, and care. Although developed primarily for screening in the low- and middle-income countries, it remains applicable for our Cascade framework.

This Cascade has been developed primarily for application within an organized screening setting for average-risk individuals, where the program develops and manages implementation of cervical screening guidelines. In this scenario, the program is also responsible for the invitation and recall of WIC, testing and diagnosis, and management of follow-up. In addition, an organized screening program has established quality control processes for evaluating performance [28]. Quality control through all program levels can substantially improve outcomes and success of the screening program.

In the Cascade, we introduce “uptake” as a measure; however, it is important to note that uptake at a new stage in the Cascade typically indicates retention from a prior stage. In a cross-sectional analysis, screening programs can look at a specific point in time and evaluate the effectiveness and performance of their program (e.g., where attritions or loss to follow-up occur). This facilitates the identification of care stages with the lowest retention rates, enabling targeted resource allocation or reallocation, and the mitigation of loss to follow-up at subsequent care. The Cascade can be applied cross-sectionally and longitudinally to monitor program uptake and retention across the care pathway, and over time at each stage.

## 3. Cervical Cancer Screening Cascade Stages

**Figure 1:**
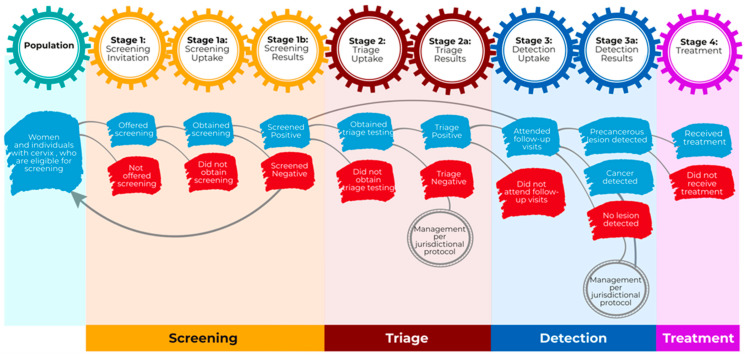
Cervical Cancer Screening Cascade. 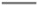 Lines represent the anticipated flow between stages in the cascade of a cervical cancer screening program and indicate that the proportion of patients in a given stage is calculated from the number of patients in the preceding stage with available data. 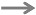 Arrows denote a return to the main population pool.

The Cervical Cancer Screening Cascade outlines four main phases: Screening, triage, detection, and treatment. Each primary stage includes two substages, emphasizing “uptake” and “results.” The Screening stage has one additional substage: “screening invitation” (Figure 1 and Table 2).

### 3.1. Population of Interest

**Figure 2:**
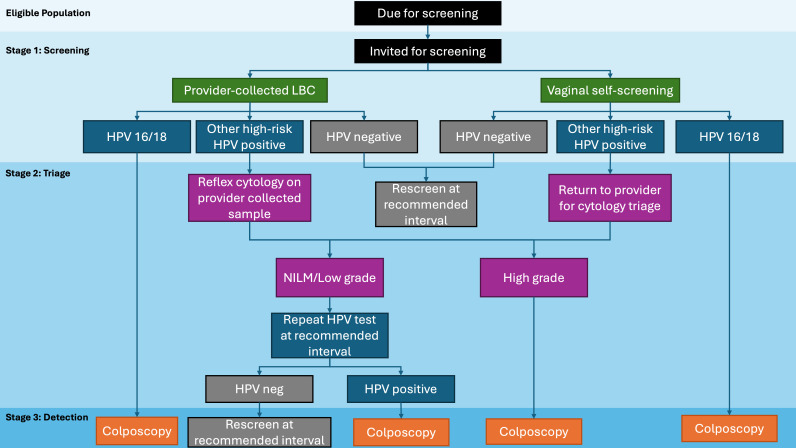
Fictional HPV primary screening program (Program X).

The Cascade was developed to be applicable to populations of WIC that are eligible for average-risk, routine cervical screening. To allow for illustration of the Cascade, a fictional organized Cervical Screening Program, hereafter referred to as Program X, that has implemented HPV primary screening has been developed (Figure 2). In fictional Program X, WIC are eligible for cervical screening if due for screening and between the ages of 25 and 70 years. In Program X, a screened individual may have their sample taken by a clinician (cervical or vaginal) or collect a vaginal sample themselves.

### 3.2. Screening

#### 3.2.1. Stage 1: Screening Invitation

Stage 1 of the Cervical Cancer Screening Cascade begins with assessing the screening reach rate—the proportion of eligible WIC who are invited to or offered screening. This rate helps estimate how effectively the program is reaching its target population. By monitoring both the screening reach rate and the missed rate (calculated as 1—screening reach rate), healthcare teams can identify gaps in infrastructure (e.g., failure to send or deliver invitations) and facilitate outreach to WIC who have not received invitations.

#### 3.2.2. Stage 1a: Screening Uptake

In Stage 1a, evaluation of screening uptake involves determining the proportion of invited WIC who have undergone cervical screening (Table 2). In Program X, HPV primary screening is offered via provider-collected (cervical) or patient self-collected (vaginal).

Monitoring the screening participation rate will identify gaps in reaching the WHO 70% and CPAC 90% screening targets. If possible, analyzing the demographics of invited WIC who do not undergo screening (screening attrition rate, calculated as 1—screening participation rate) may facilitate targeted interventions to increase participation.

#### 3.2.3. Stage 1b: Screening Results

In Stage 1b, the screening positivity rate is introduced, representing the proportion of screened WIC with a positive test result. For those who are HPV positive, the positivity rate can be further analyzed by other factors routinely collected by a jurisdiction, such as age, geographic location (for example, postal code), and screening history, to offer additional insights [27]. In Program X, those who screen negative return to routine screening and are reclassified as the “population eligible for screening”. This group will be invited for recall at the time interval determined by the screening program. In Program X, partial genotyping is available; as a result, management of positive results depends on the detected HPV genotype.

### 3.3. Triage

#### 3.3.1. Stage 2: Triage Uptake

With HPV primary screening, a triage test is required to improve specificity of HPV testing [29]. Triage testing utilizes risk-based management of cervical precancer following positive primary screening results, minimizing the chances of over-referral to diagnostics and treatment. The choice of triage method depends on the primary test conducted. In Program X, an HPV 16/18 positive screen will bypass triage and proceed directly to colposcopy referral, whereas a positive result for other high-risk (pooled) types of HPV necessitates cytology triage testing (provider-collected if HPV test was self-collected; or automatic triage to cytology at the laboratory if HPV was collected by a provider with liquid-based cytology).

The triage completion rate measures the proportion of screen-positive WIC referred to triage who completed all required triage visits out of the total number of WIC referred to triage. This metric is crucial for assessing the triage uptake rate from screening to triage testing. Understanding factors affecting triage attrition rate, calculated as 1—triage completion rate, can allow for targeted efforts to improve triage rates.

#### 3.3.2. Stage 2a: Triage Results

In an HPV primary screening program, recommendations for scheduled follow-up depend on the combined HPV and triage results (Figure 2). WIC identified as high-risk due to combined positive primary screen and triage results progress to the detection stage for diagnostic testing, which is typically a referral to colposcopy. In Program X, WIC with other high-risk HPV-positive results who then receive cytology triage testing with high-grade squamous intraepithelial lesions (HSIL) or greater are recommended for colposcopy.

Monitoring the triage positivity rate is essential for determining the necessity for further examinations and preventing unnecessary over-testing and over-treatment [30].

### 3.4. Detection

#### 3.4.1. Stage 3: Detection Uptake

The primary aim of any cervical screening program is to detect pre-cancerous lesions, in order to facilitate treatment. As such, follow-up after a positive test result is crucial. It enables early detection of precancerous lesions and facilitates timely treatment to prevent progression to cancer, or confirmation of a cancer diagnosis requiring further treatment. In most jurisdictions, including Program X, the detection stage will typically involve colposcopy, with or without a biopsy.

The detection uptake stage introduces a measure of follow-up completion rate, defined as the proportion of WIC with a screen or triage result that requires follow-up who attend all their recommended diagnostic follow-up visits (e.g., colposcopy with or without biopsy). Adherence at the detection uptake stage is critical, as loss to follow-up at this stage poses significant barriers to reducing cervical cancer incidence. Loss to follow-up, particularly for colposcopy, can result in undetected and potentially untreated precancerous lesions and an elevated risk of progression to cervical cancer [31].

#### 3.4.2. Stage 3a: Detection Results

Both attendance at colposcopy (Detection uptake) and results of diagnostic assessments are essential for determining the next steps for the individual in the screening cascade, including the possible detection of precancerous lesions, cancer, or the absence of lesions, each of which require different management. If left untreated, CIN2 or greater (CIN2+) may progress to invasive cancer. WIC diagnosed with a precancerous lesion or cancer are subsequently referred for treatment. In cases where no lesion is detected, the management follows jurisdictional protocols.

Estimating the proportion of precancerous lesions among WIC who attended their follow-up visits at the detection stage is referred to as the precancer rate and is essential for monitoring and ensuring timely referral and access to treatment.

### 3.5. Treatment

#### Stage 4: Treatment Uptake

WIC are referred for treatment upon the detection of a precancerous lesion, and the WHO recommends initiating treatment within 6 months of detection [2]. Treatment with either cold knife conization or large loop excision of the transformation zone (LEEP) is required for CIN2+, while CIN1 lesions typically do not necessitate treatment unless they persist or progress [32].

In the quest to eliminate cervical cancer, the WHO has set a goal of achieving a 90% treatment rate among WIC identified with precancerous lesions [2]. The treatment rate is defined as the proportion of WIC with detected lesions who have received treatment. Since the goal of screening is to identify and treat lesions before they progress to cancer, we propose excluding WIC with a historical cancer diagnosis or those who have undergone invasive treatments, such as surgery (e.g., hysterectomy), chemotherapy, or radiation therapy, when measuring treatment rate. A high treatment rate contributes to improved outcomes in screening programs by reducing cervical cancer incidence and mortality rates.

## 4. Practical Steps for Using the Cervical Cancer Screening Cascade

The Cascade is designed to improve program evaluation by providing a structured approach to monitor uptake and retention along the screening and treatment pathway and guide emerging programs in the development of robust data collection and reporting infrastructures. The following steps outline how programs can effectively use the Cascade to achieve these goals, particularly by addressing data gaps and optimizing care pathways:

1. Identify the Population:
  • Define the eligible group for cervical cancer screening (e.g., age range, risk factors, or geographic location).
  • Ensure you have access to data on the number of eligible WIC and the corresponding stages of their care.
2. Map Data onto the Cascade Stages:
  • Break down the screening algorithm and categorize available data into the four key stages of the Cascade:
    i. Screening: Number of WIC invited, participating, and screened.
    ii. Triage: Individuals completing triage testing after an abnormal result, and number of those with positive triage results.
    iii. Detection: Individuals undergoing follow-up diagnostic testing, and number diagnosed with precancerous lesions.
    iv. Treatment: Number receiving appropriate treatment.
  • Identify gaps in data or data collection system at each stage and identify strategies to address them.
3. Calculate Key Metrics:
  • Use program data to calculate the corresponding measures at each stage of the Cascade. Example: screening participation rate = (number screened ÷ eligible population) × 100
  • Calculate the attrition rate or loss to follow-up rate at each stage. Example: triage attrition rate = 1—triage completion rate
4. Compare Metrics with Targets:
  • Assess whether the calculated metrics meet established benchmarks (e.g., WHO targets of 70% screening participation and 90% treatment rates).
  • Identify any deviations from the targets and determine potential areas for improvement.
5. Identify Attrition Points:
  • Look for stages where the largest attrition or loss to follow-up occurs (e.g., WIC who test positive but do not proceed to triage).
  • If needed, conduct a more granular-level or stratified analysis to gather more insight.
  • Analyze reasons for attrition, such as lack of accessibility, awareness, or follow-up mechanisms.
6. Implement Targeted Interventions:
  • Develop strategies to address gaps identified at each stage.
  • Example: If triage completion rates are low, consider introducing automated reminders or mobile diagnostic units to improve access.
7. Monitor Progress over Time:
  • Use the Cascade framework longitudinally to track program improvements and the impact of interventions.
  • Compare data across different years or regions to identify trends and share best practices.

## 5. Discussion

This paper proposes a comprehensive Cervical Cancer Screening Cascade designed to track the process from initial invitation through to follow-up care. It is also designed to serve as a foundational framework for monitoring, evaluating, and strengthening HPV primary screening. The primary objective is to enable programs to monitor progress, enhance participation, and improve retention at each stage, while facilitating the early detection and treatment of precancerous lesions to prevent progression to invasive cancer and achieve the WHO and CPAC cervical cancer elimination goals. We propose two main actionable purposes for the Cascade:

1. A guiding framework for emerging programs: Screening programs in developmental stages or those undergoing transitions, such as the shift from cytology to HPV screening, can utilize this cascade to embed clearly defined touchpoints with automated data capture through integration with electronic health records or laboratory information systems. The framework supports the development of robust data collection and reporting infrastructures, essential for optimizing program effectiveness.
2. A standardized evaluation and benchmarking tool: The cascade provides a consistent and systematic approach for collecting and reporting metrics across key stages of screening. By adopting this standardized framework, existing programs can better evaluate and report their performance, facilitate cross-program comparisons, and share insights and best practices to collectively advance cervical cancer prevention efforts.

This dual-purpose framework may not only address gaps in data tracking and reporting but also equip programs with the tools needed to build capacity, improve infrastructure, and enhance outcomes at every stage of the Cervical Cancer Screening Cascade. Although not the primary purpose of the Cascade, it can also be used to examine specific populations or groups who may face unique barriers to care. For example, where postal code data are available, programs can explore screening patterns across urban and rural settings. Similarly, metrics can be analyzed by age or screening history (e.g., first-time vs. regular screeners), provided that such demographic data are collected within the screening program. This may allow for implementation of targeted interventions addressing populations with the highest rates of precancer and cancer.

The Cascade can serve not only as a tool for tracking progress but also as a means of guiding targeted interventions, improving program transparency, and fostering cross-program collaboration to optimize cervical cancer prevention efforts (e.g., enhancing screening uptake, triage completion, and treatment rates). To maximize the utility of the Cervical Cancer Screening Cascade, we advocate for the inclusion of more comprehensive metrics, particularly for the triage and treatment stages, to enable more robust and holistic program evaluations. Although fictional “Program X” implemented vaginal self-sampling and cytology as the triage tool, alternative methods of self-sampling (ex: urine collection) and alternative options for triage (ex: extended genotyping or methylation), are possible scenarios in current or future programs. This Cascade has been developed to allow for flexibility and variability in collection and or triage strategies, while still measuring retention at the various stages.

The Cascade is designed to improve program evaluation by providing a structured approach to monitor uptake and retention along the screening and treatment pathway and guide emerging programs in the development of robust data collection and reporting infrastructures. Programs can begin by clearly identifying the eligible population, such as women aged 30–49 years living in underserved regions, and ensuring access to data that tracks WIC across each stage of care. Moreover, by calculating stage-specific metrics, such as participation rates (e.g., the proportion of eligible WIC screened) and attrition rates (e.g., the proportion who fail to proceed from triage to diagnosis), programs can evaluate their performance against established benchmarks, such as the WHO’s 70% screening and 90% treatment targets.

In cases where benchmarks are not met, targeted interventions may be necessary. For instance, if a program finds that only 50% of WIC with positive screening results complete triage, identification of interventions specifically at triage uptake may be required. For example, introducing SMS reminders or incorporating a patient navigator into the screening program to facilitate attendance at follow-up could help close the gap. This Cascade has been developed for application within a high-resource setting. We recognize that application within lower-resourced settings would require modification of the Cascade. To utilize the Cascade within such settings would likely require re-design of the Cascade to adapt to the particular setting. For example, in a program that utilizes HPV self-collection, with referral to VIA and thermocoagulation for those HPV positive, a program may need to collapse stages 2–4 (triage to treatment). As more low-resourced settings implement HPV screening as screen-and-treat or point-of-care models, development of a tailored Cascade for such programs could prove valuable.

Building on similar principles to the Cascade, a recent study examined loss to follow-up at different stages of the Dutch HPV-based cervical cancer screening program [6]. The Dutch study focuses specifically on loss to follow-up (calculated as 1—participation rate or 1—completion rate) rather than uptake and adopts a more granular approach. It calculates loss to follow-up at each point in the pathway using multiple metrics within each stage—tracking four distinct metrics during triage and two during detection—allowing for a more precise understanding of where WIC are lost along the continuum of care. In contrast, the Cascade offers a high-level view of participation and completion across key stages (screening, triage, detection, treatment) and reports uptake or completion rates at each of these stages as a whole. For instance, we report a single “triage completion rate”, reflecting the proportion of WIC who complete all required triage visits. Additionally, the Dutch study incorporates stratified analyses by age, sampling method, region, and screening history, offering deeper insights into attrition trends [6]. This detailed, moment-specific analysis complements the Cascade’s stage-level framework by highlighting nuances that may be overlooked in broader assessments. While we recommend beginning with stage-level evaluations to identify critical gaps, adopting such granular analyses as a secondary step could enhance its ability to pinpoint weaknesses, inform targeted interventions, and optimize care pathways.

## 6. Conclusions

Cervical cancer remains a significant global health challenge, but its elimination is possible where regions are able to meet the targets established by the WHO and national bodies, such as CPAC. As regions transition to implementation of HPV primary screening, comprehensive evaluation protocols are required to measure screening and treatment targets. Adopting a comprehensive evaluation framework, such as this Cervical Cancer Screening Cascade, will help programs assess their elimination progress. This paper introduced a comprehensive framework designed to track the process from initial invitation through treatment and evaluate cervical cancer screening programs along the care pathway. By defining four key stages: screening, triage, detection, and treatment, each with specific substages focused on ‘uptake’ and ‘results,’ the Cascade provides a structured model for tracking program progress and effectiveness in eliminating cervical cancer.

The proposed Cascade serves as both a guiding framework for emerging programs and a standardized evaluation and benchmarking tool for existing programs. It enables programs to measure success as they transition from cytology to HPV primary screening and to establish robust data collection and reporting infrastructures. The Cascade offers a systematic approach to evaluate performance, compare outcomes across initiatives, and share best practices to collectively advance cervical cancer prevention efforts.

As cervical cancer prevention efforts advance, we call on stakeholders—policymakers, researchers, and program implementers—to adopt and refine the Cascade. This collective effort will enable programs to address gaps in data tracking and reporting, build capacity through cross-program learning and targeted interventions, and improve outcomes at every stage of the screening process. By fostering collaboration, cross-program learning, transparency, and innovation, these initiatives have the potential to transform cervical cancer from a persistent health challenge into a preventable and manageable condition, bringing us closer to the goal of elimination.

## Figures and Tables

**Table 1:** Cervical cancer care cascade studies.

Study	Target Population	Age Range	Primary Screening Method	Measures
Grover et al. (2023) [25]	Women receiving radiation therapy in Botswana	≥27 years	Not specified	Treatment delay Survival rates
Taghavi et al. (2022) [22]	Women living with HIV in Zimbabwe	≥18 years	Visual inspection with acetic acid and cervicography (VIAC)	Screening uptake Screening result Treatment uptake Rescreening uptake Rescreening results
Garcia et al. (2022) [26]	Women in rural Guatemala	21–65 years	Cytology	Screening uptake Screening result Triage + Detection uptake Treatment uptake
Rohner et al. (2021) [23]	Women living with HIV in South Africa	≥18 years	Cytology/ Pap smear	Screening Results Follow-up uptake Treatment uptake
Wang et al. (2019) [24]	Rural Chinese women	35–64 years	Cytology/ Pap smear	Screening uptake Screening results Detection uptake Detection result

**Table 2:** Cervical cancer screening cascade definitions.

Stage	Measure	Numerator	Denominator
**Screening**			
**Stage 1: Screening Invitation**	Screening Reach Rate	Number of WIC invited to screen	Eligible WIC: WIC eligible for average risk, routine screening, within eligible screening age range
**Stage 1a: Screening Uptake**	Screening Participation Rate	Number of screened WIC (self-sampled and/or provider-collected)	Number of invited WIC
**Stage 1b: Screening Results**	Screening Positivity Rate	Number of WIC with abnormal results that refer to triage or diagnostic follow-up	Number of screened WIC
**Triage**			
**Stage 2:** **Triage Uptake**	Triage Completion Rate	Number of WIC referred to triage who completed all required triage visits	Number of WIC referred to triage
**Stage 2a: Triage Results**	Triage Positivity Rate	Number of WIC with positive triage results	Number of WIC completing triage
**Detection**			
**Stage 3: Detection Uptake**	Follow-up Completion Rate	Number of WIC completing diagnostic follow-up visits	Number of WIC referred to diagnostic follow-up (directly after primary test or following triage)
**Stage 3a: Detection Results**	Precancer Rate	Number of WIC with precancerous lesions	Number of WIC completing diagnostic follow-up visits
**Treatment**			
**Stage 4: Treatment Uptake**	Treatment Rate	Number of WIC with detected precancerous lesions who received treatment	Number of WIC with detected precancerous lesions

WIC, women and individuals with cervices; Provider-collected, cervical or vaginal collection; self-sampled, vaginal self-collection.
